# Supplementary figures and images for: Modulating tumor immunity using advanced microbiome therapeutics producing an indole metabolite
Source: EMBO Rep. 2025 Mar 7;26(7):1688–708. doi: 10.1038/s44319-025-00386-9 (PMC11977207; doi:10.1038/s44319-025-00386-9)

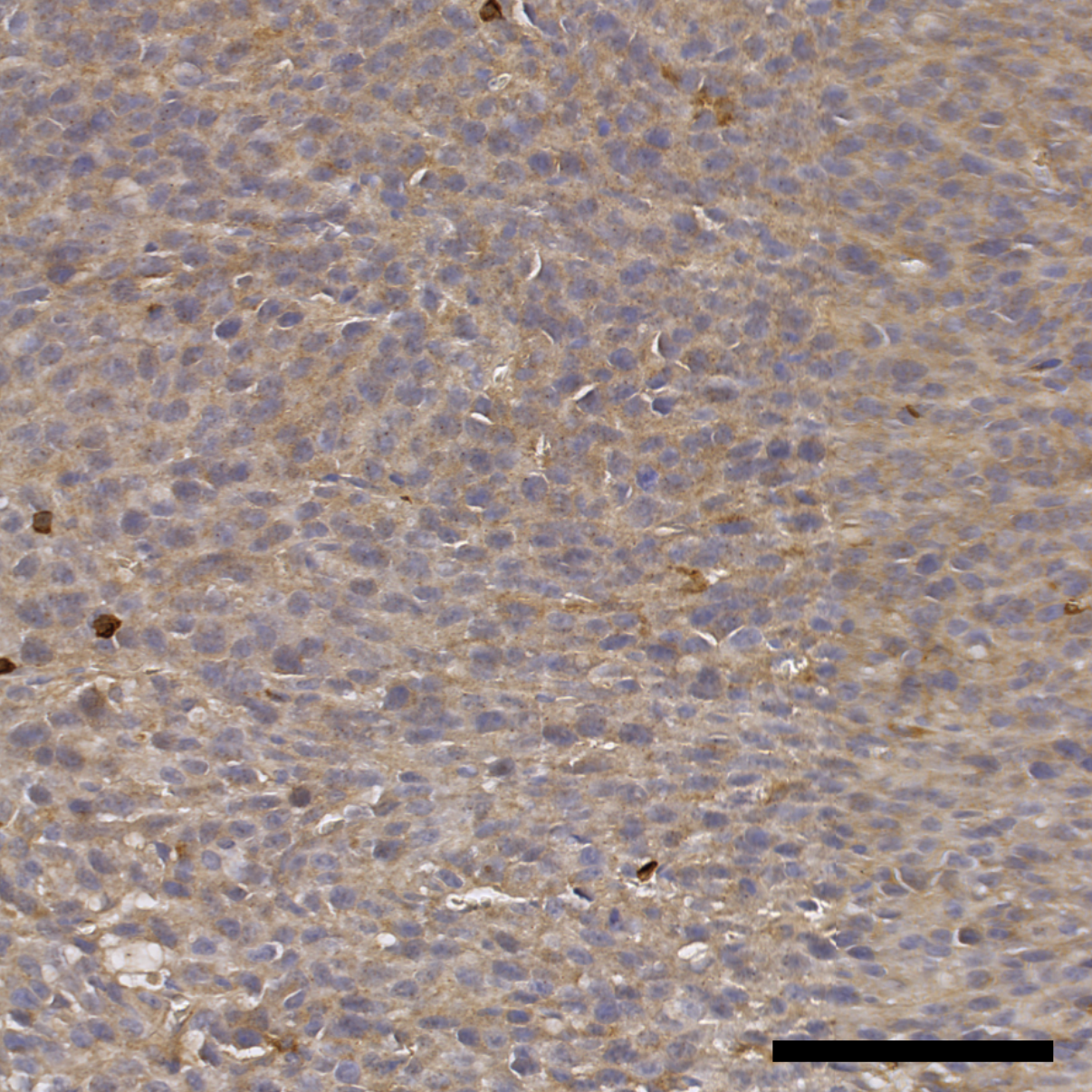

Supplement: Supplementary file 4 — Source data Fig. 3 [file 44319_2025_386_MOESM4_ESM.zip › Figure 3/EcN_Ctrl_CD4+.tiff]

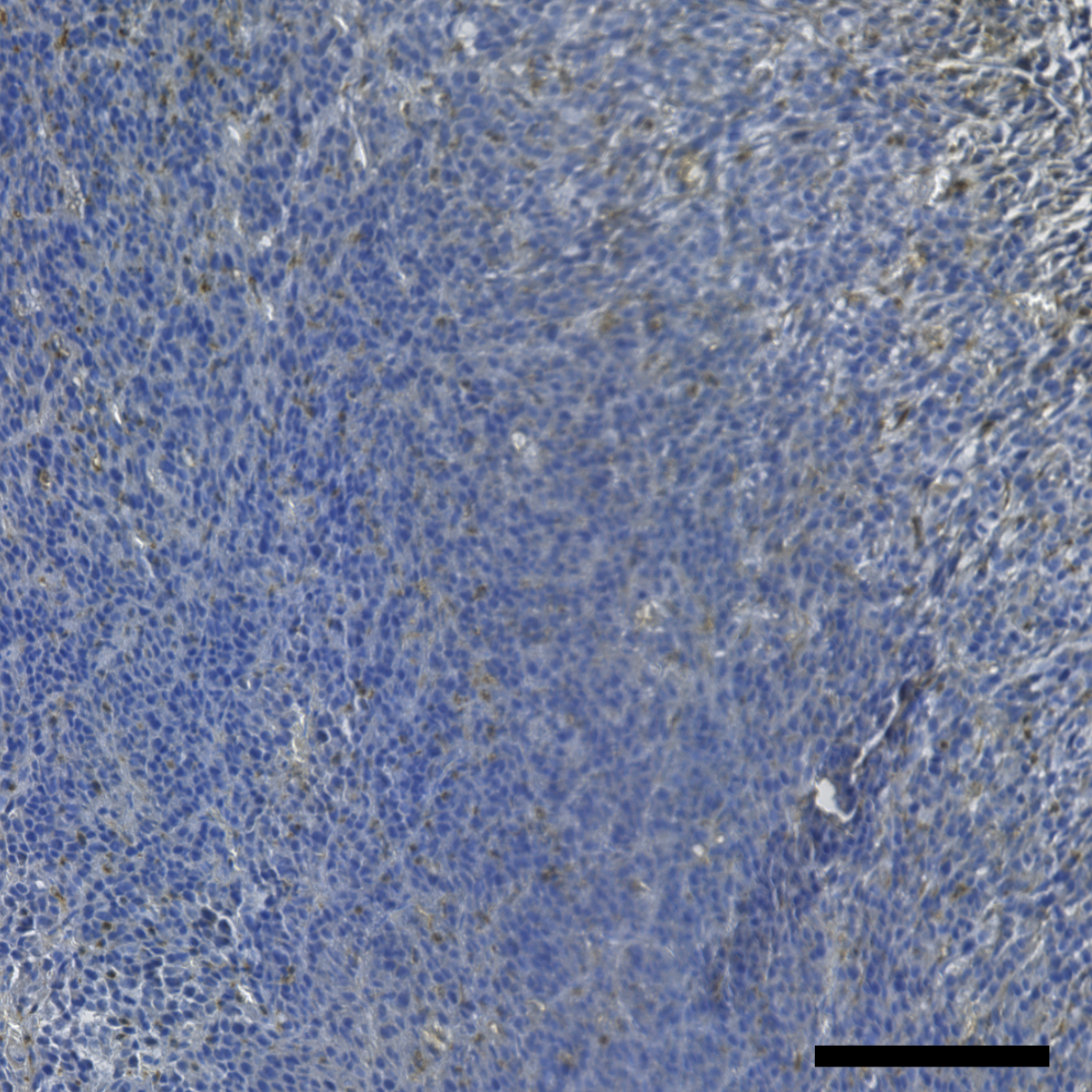

Supplement: Supplementary file 4 — Source data Fig. 3 [file 44319_2025_386_MOESM4_ESM.zip › Figure 3/EcN_Ctrl_CD68+.tiff]

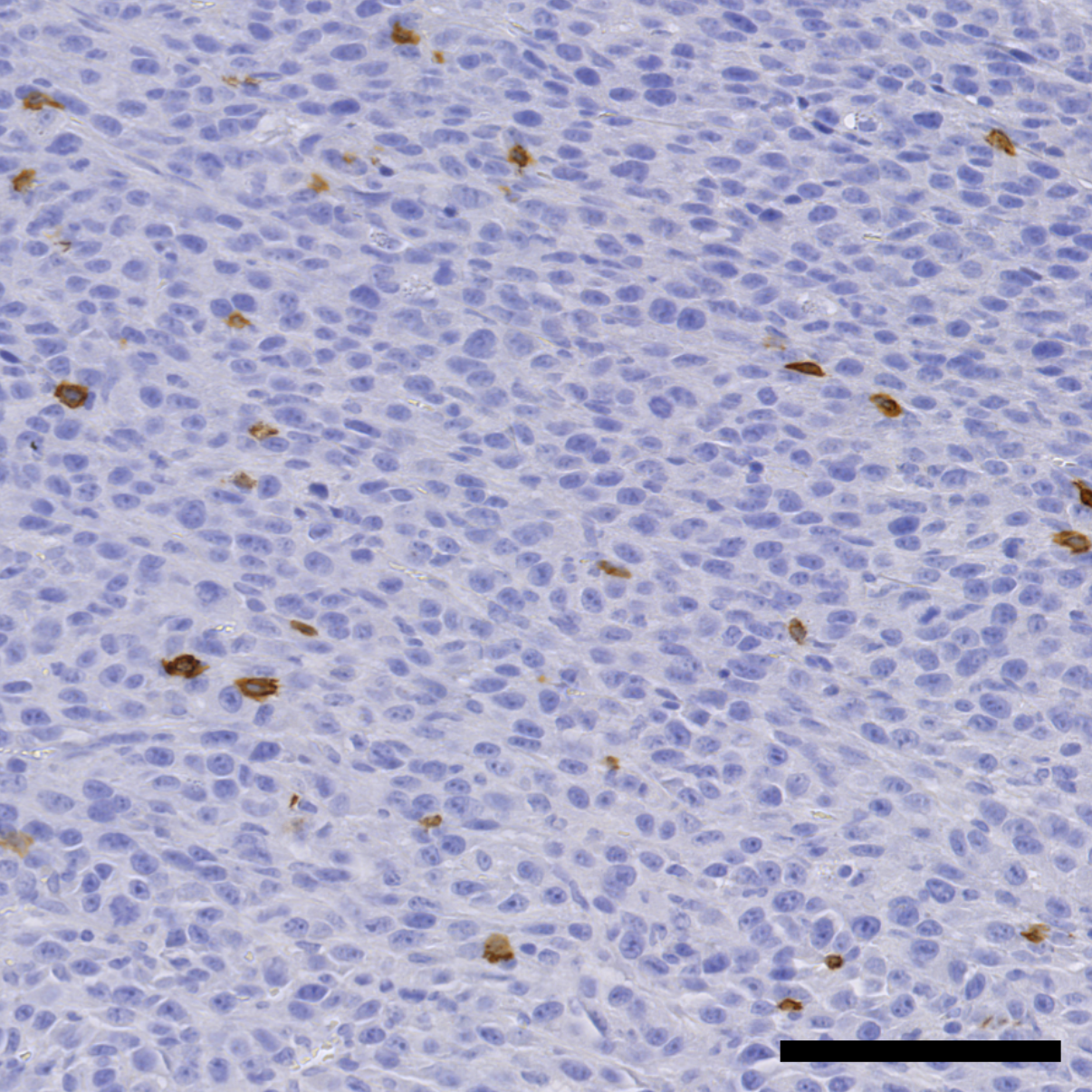

Supplement: Supplementary file 4 — Source data Fig. 3 [file 44319_2025_386_MOESM4_ESM.zip › Figure 3/EcN_Ctrl_CD8+.tiff]

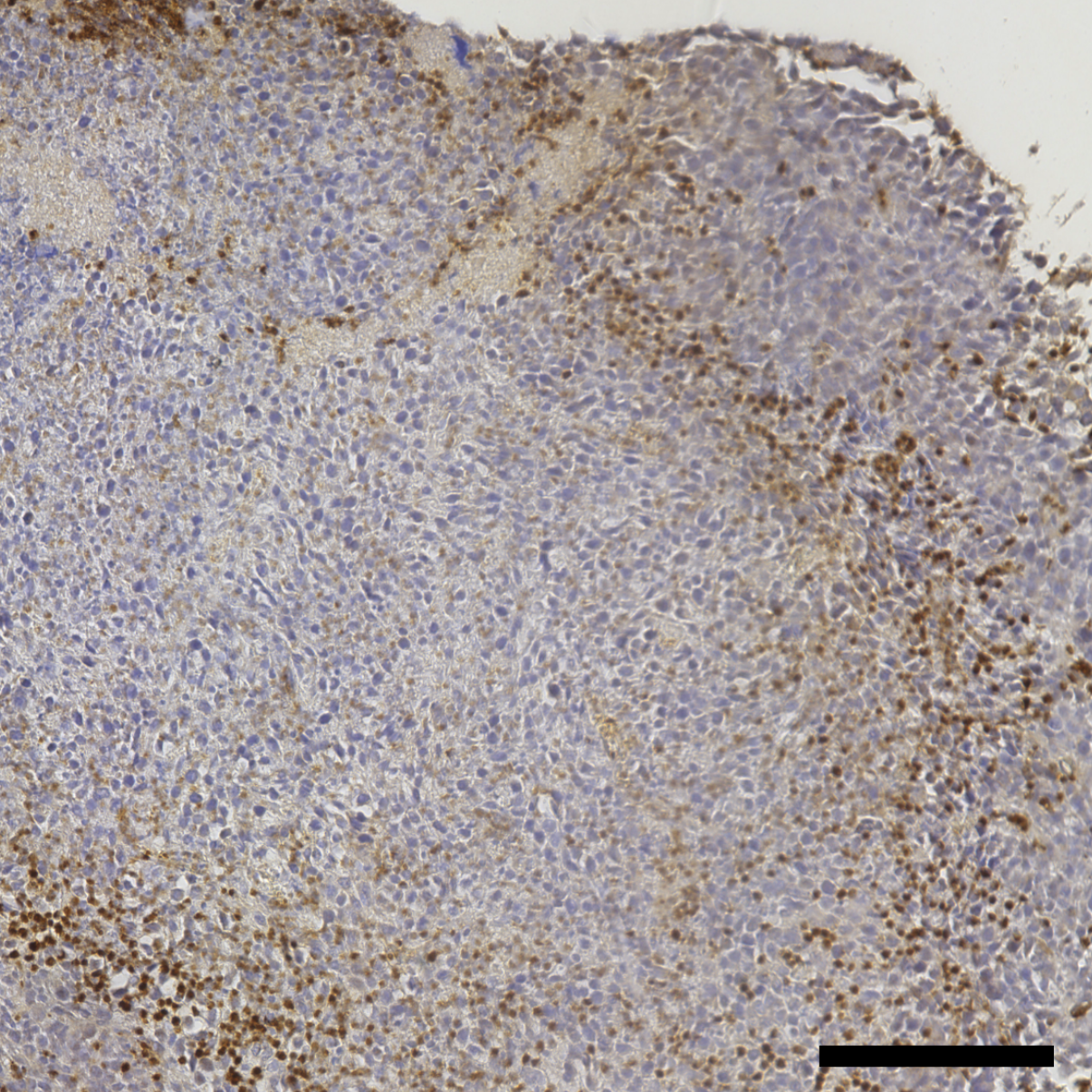

Supplement: Supplementary file 4 — Source data Fig. 3 [file 44319_2025_386_MOESM4_ESM.zip › Figure 3/EcN_Ctrl_ELA2.tiff]

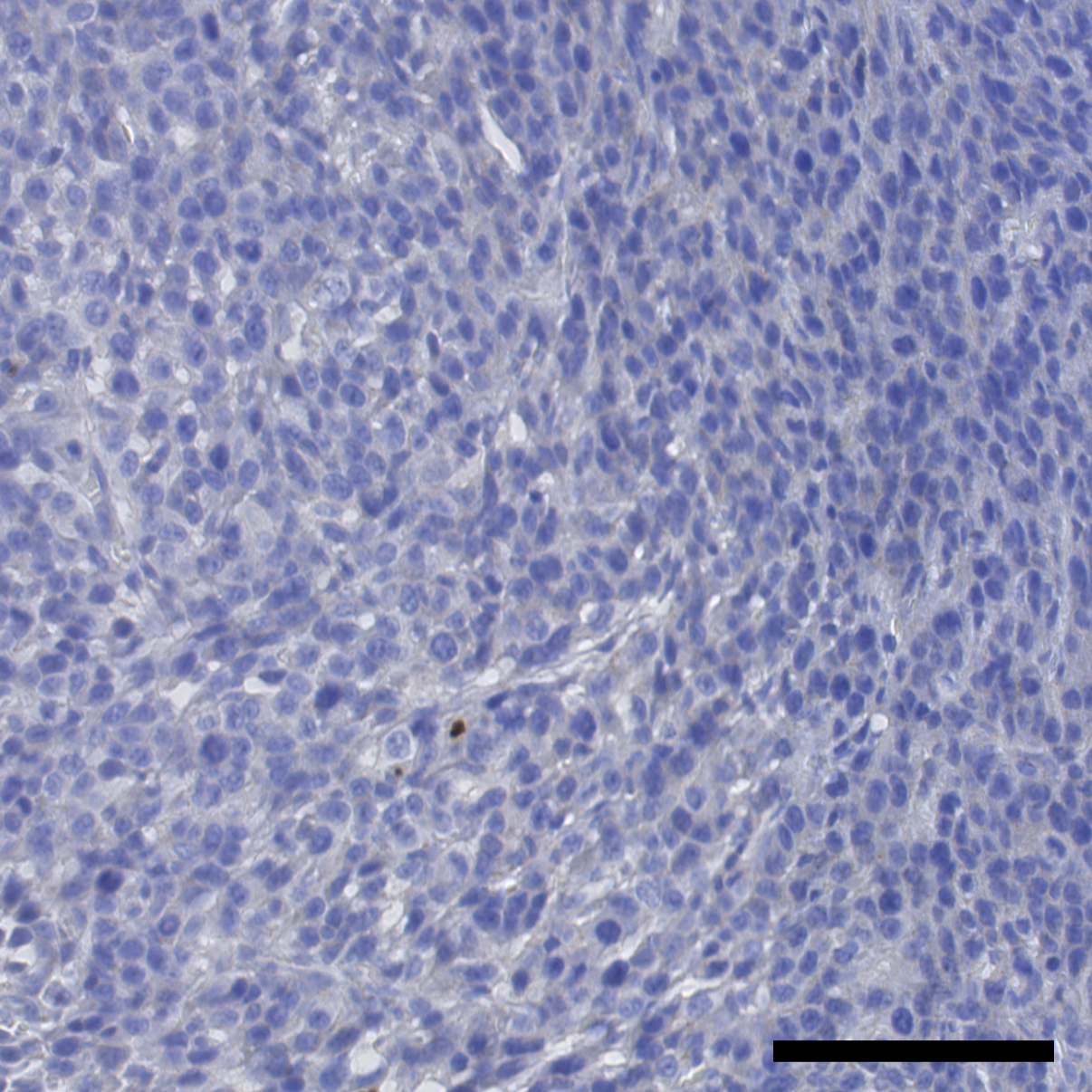

Supplement: Supplementary file 4 — Source data Fig. 3 [file 44319_2025_386_MOESM4_ESM.zip › Figure 3/EcN_Ctrl_FOXP3+.tiff]

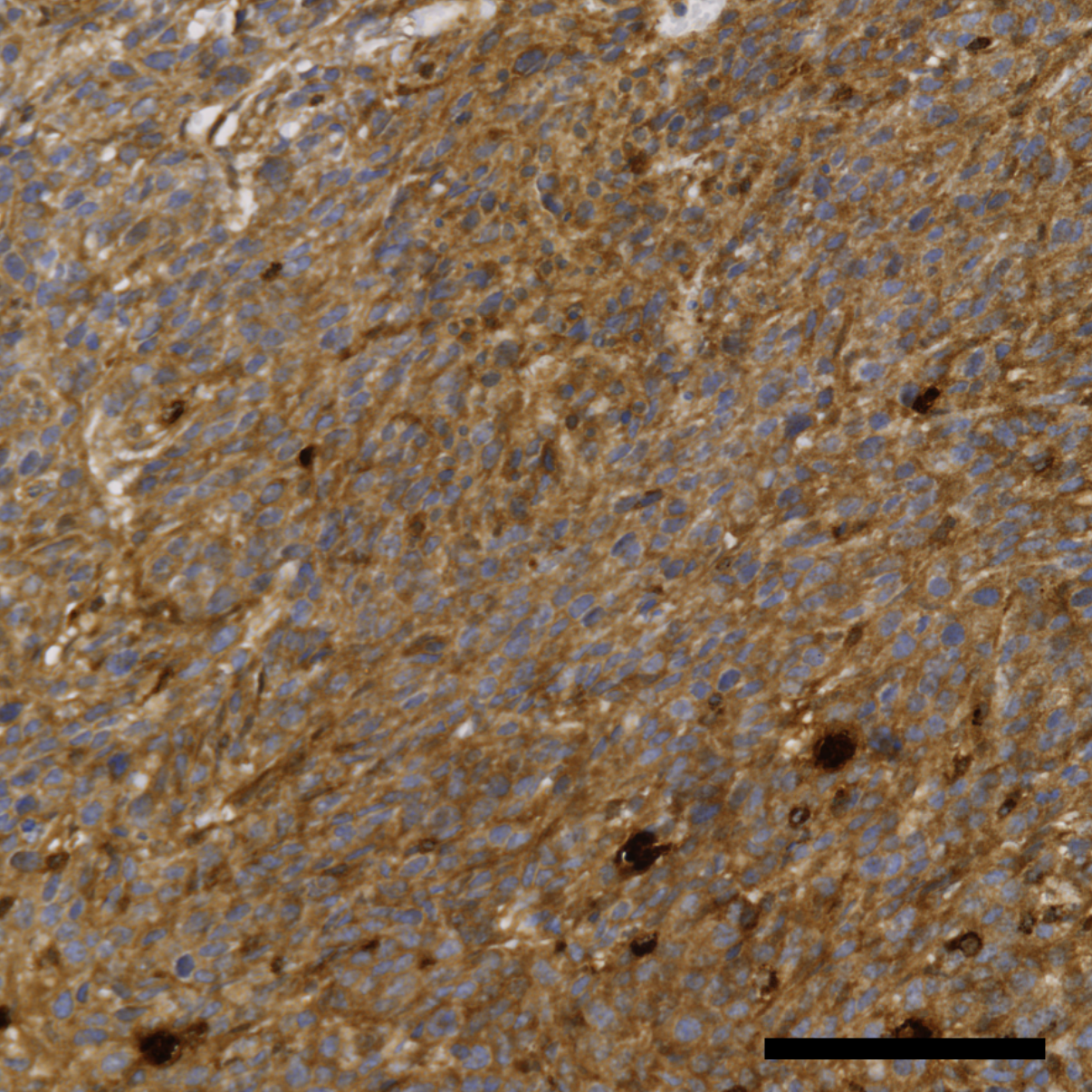

Supplement: Supplementary file 4 — Source data Fig. 3 [file 44319_2025_386_MOESM4_ESM.zip › Figure 3/EcN_Ctrl_GrzB+.tiff]

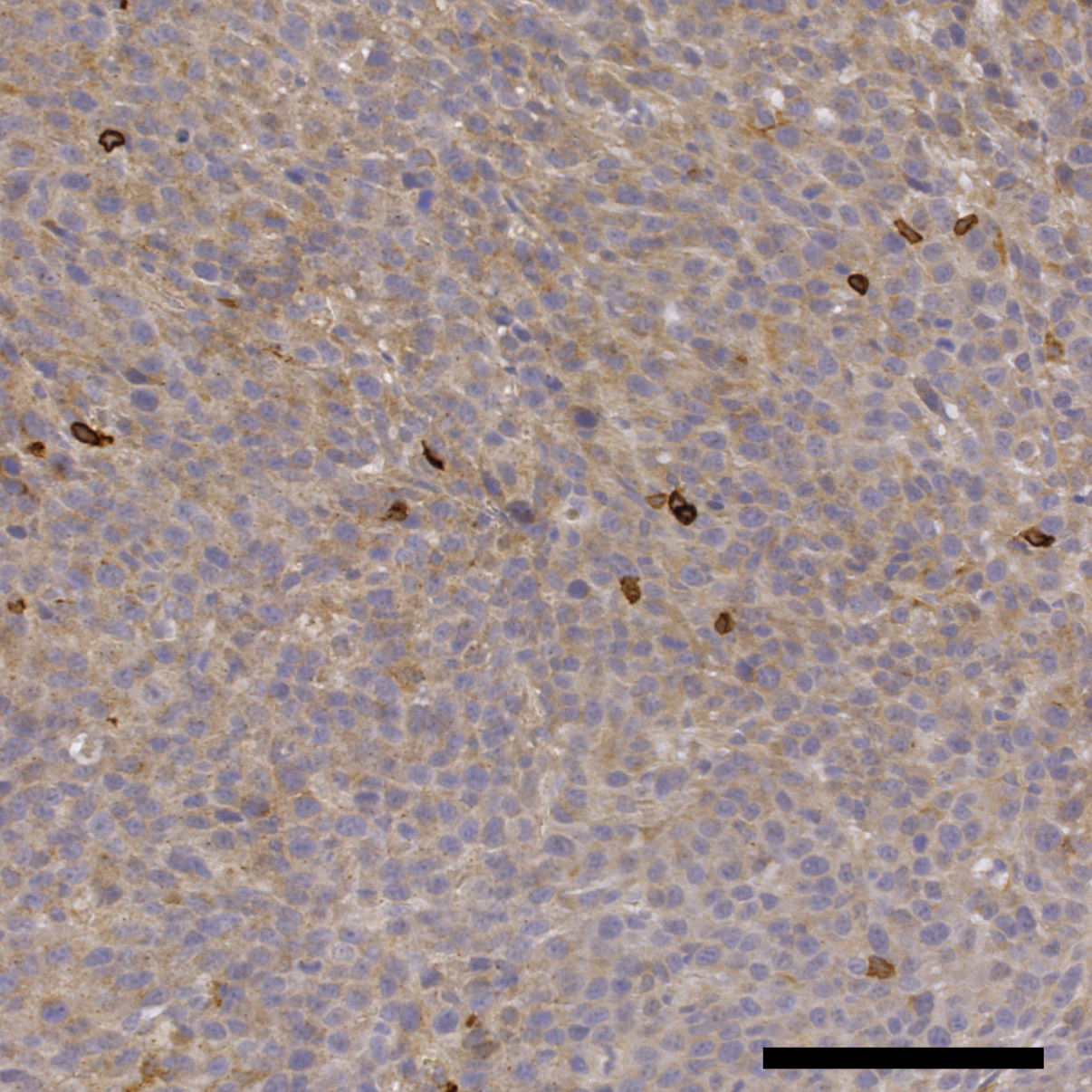

Supplement: Supplementary file 4 — Source data Fig. 3 [file 44319_2025_386_MOESM4_ESM.zip › Figure 3/EcN_IAA_CD4+.tiff]

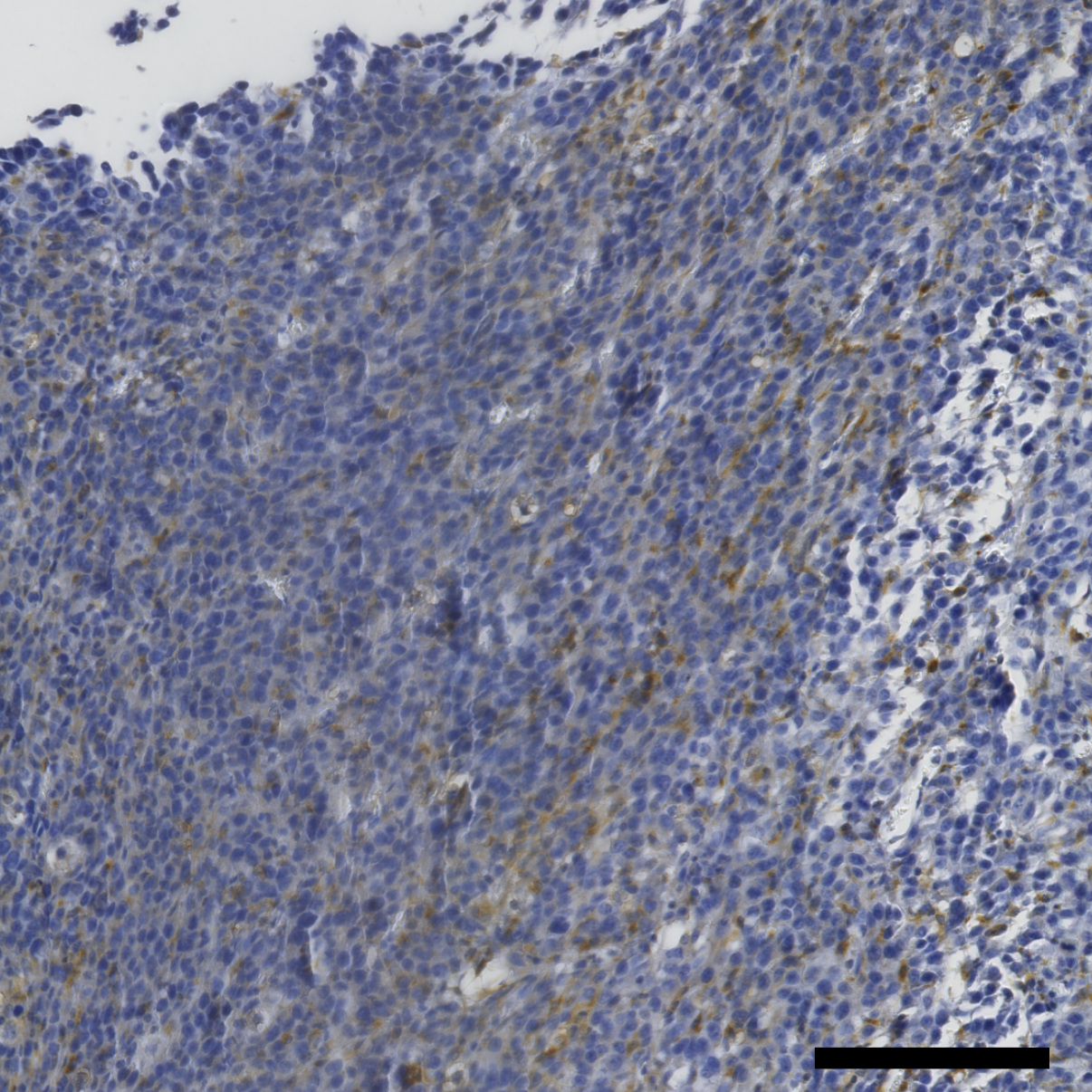

Supplement: Supplementary file 4 — Source data Fig. 3 [file 44319_2025_386_MOESM4_ESM.zip › Figure 3/EcN_IAA_CD68+.tiff]

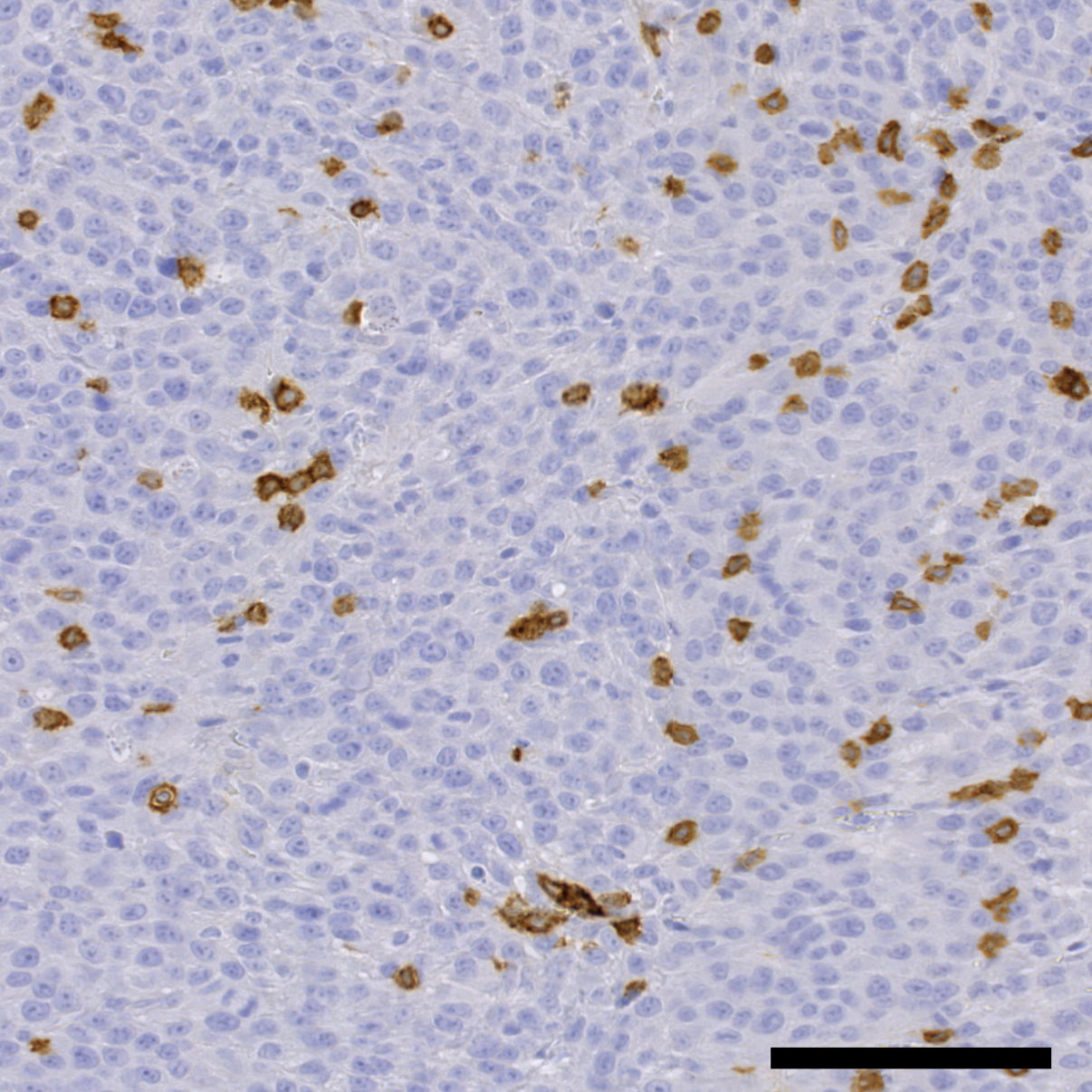

Supplement: Supplementary file 4 — Source data Fig. 3 [file 44319_2025_386_MOESM4_ESM.zip › Figure 3/EcN_IAA_CD8+.tiff]

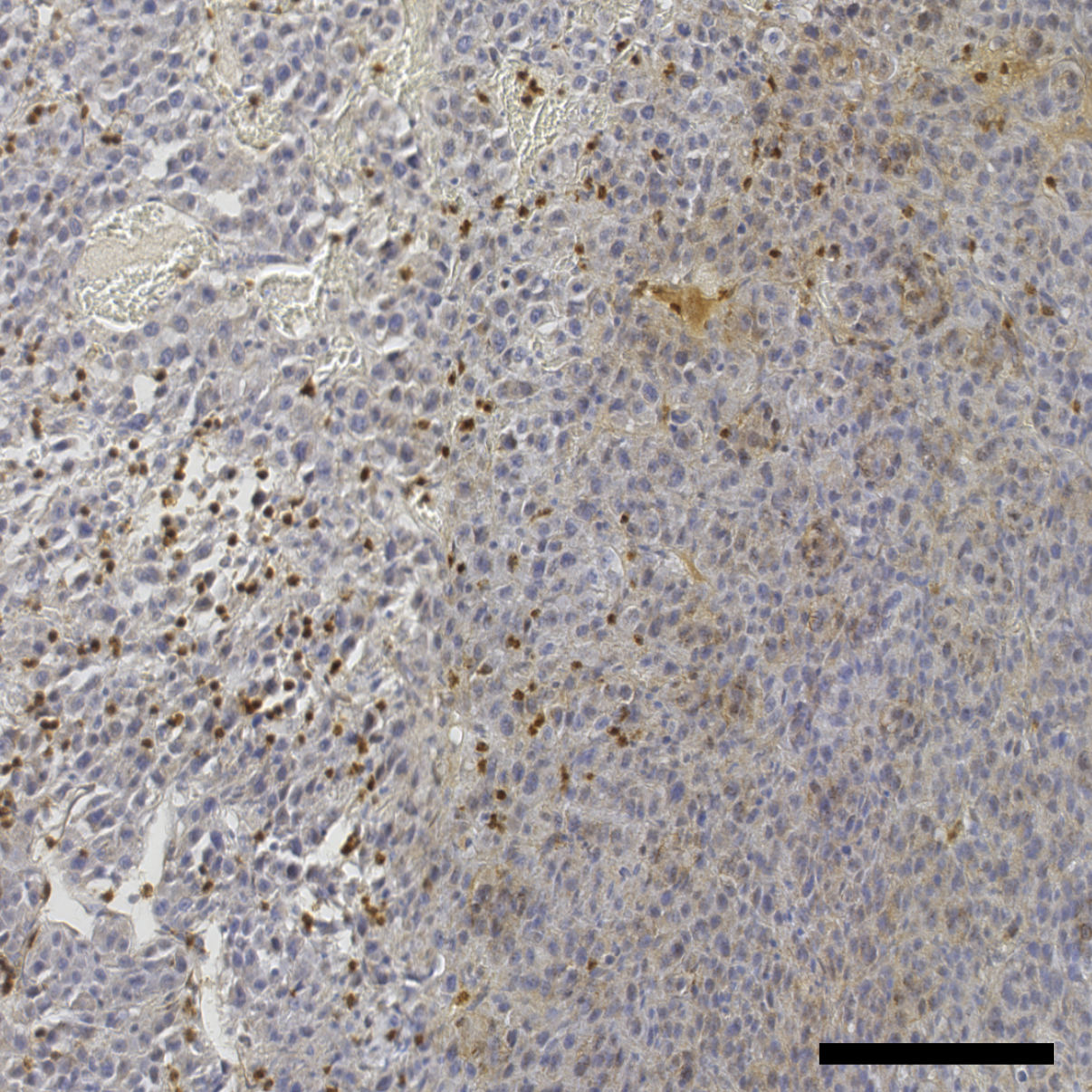

Supplement: Supplementary file 4 — Source data Fig. 3 [file 44319_2025_386_MOESM4_ESM.zip › Figure 3/EcN_IAA_ELA2.tiff]

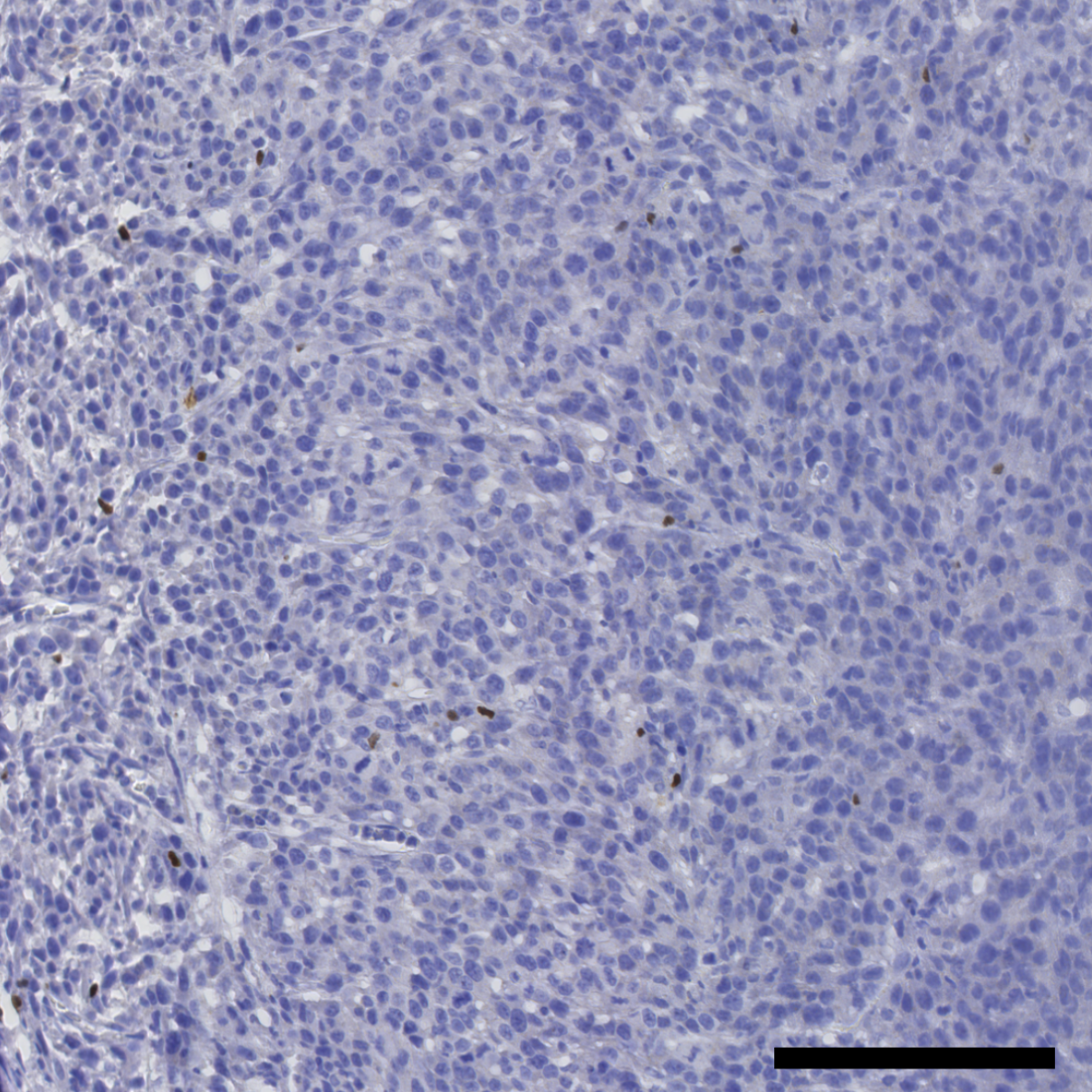

Supplement: Supplementary file 4 — Source data Fig. 3 [file 44319_2025_386_MOESM4_ESM.zip › Figure 3/EcN_IAA_FOXP3+.tiff]

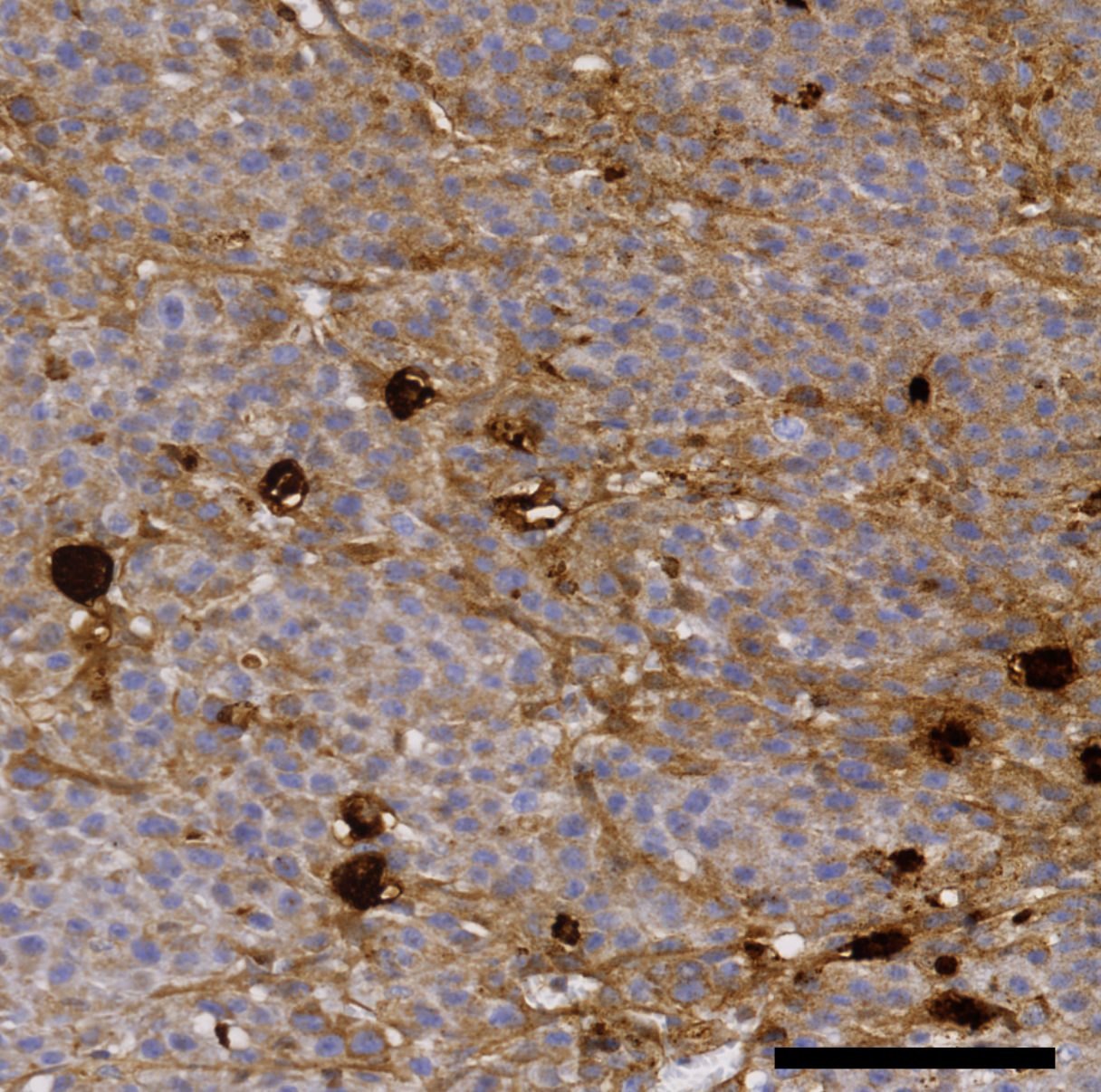

Supplement: Supplementary file 4 — Source data Fig. 3 [file 44319_2025_386_MOESM4_ESM.zip › Figure 3/EcN_IAA_GrzB+.tiff]
