# Supplementary material for: Modulating tumor immunity using advanced microbiome therapeutics producing an indole metabolite
Source: EMBO Rep. 2025 Mar 7;26(7):1688–708. doi: 10.1038/s44319-025-00386-9 (PMC11977207; doi:10.1038/s44319-025-00386-9)
Supplement: Supplementary file 8 — Expanded View Figures [file 44319_2025_386_MOESM8_ESM.pdf]

# Expanded View Figures

**Figure EV1. Schematic representation of the pMUT1 plasmid used to produce IAA.**

The plasmid contains a ColE2-like origin of replication and a kanamycin resistance cassette for selection and the Hok/Sok toxin-antitoxin system for improved stability. The genes encoding the three-step pathway were codon-optimized for *E. coli* and inserted into the plasmid as an operon, under the control of the constitutive promoter pMS6.

 © The Author(s)

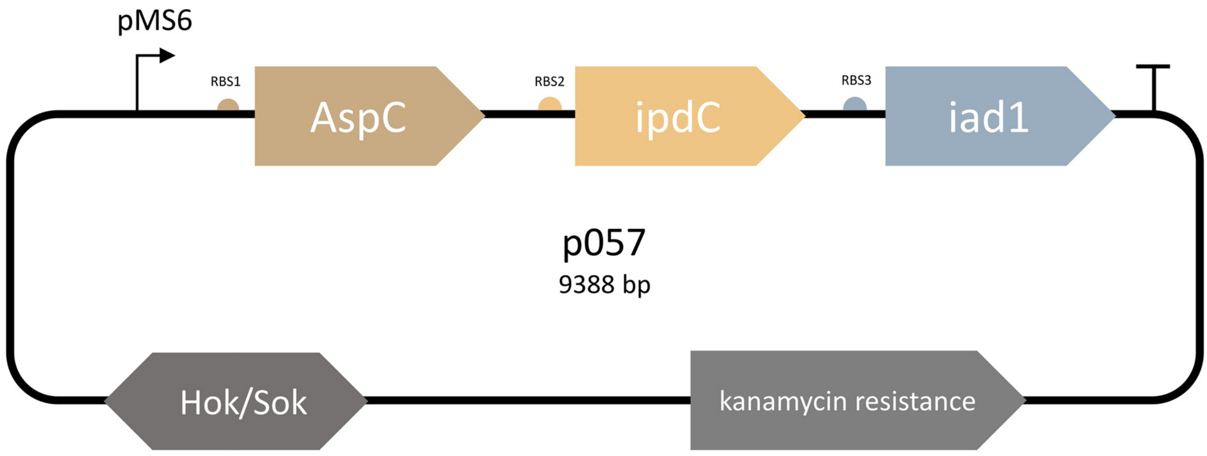


| Name | Part | Sequence (5'--> 3') |
|---|---|---|
| MS6 | Promoter | TGCTGGACTCGTCGTAATCCTGCGTGTATAATTGGC |
| RBS1 | RBS4 | AAAGGAGAA |
| AspC | Gene of interest 1 | ATGTTCGAGAATATTACTGCCGCCCCGGCTGACCCAATCTTAGGTCTTGCCGATTTGTTTCGCGCTGACGAACGGCCAGGCAAGATAAATTTGGGCATAGGCGTTTACAAGGATG<br>AAACTGGCAAGACGCCTGTCCTGACATCCGTAAAAAAGGCTGAACAATACCTTCTTGAAAACGAGACAACAAAGAACTATCTGGGGATTGATGGAATACCCGAATTTGGTCGGT<br>GCACTCAAGAACTGCTTTTCGGAAAGGGGTCTGCTCTTATCAACGATAAGCGCGCCCGTACAGCACAGACACCTGGCGGAACAGGTGCGTTGAGAGTCGCAGCGGATTTTCTGG<br>CGAAGAATACGAGCGTTAAAAGAGTGTGGGTCTCAAATCCATCGTGGCCGAACCATAAGAGCGTCTTCAACAGTGCAGGTTTAGAAGTCAGAGAGTATGCGTACTACGACGCGG<br>AAAATCATACACTGGACTTCGACGCGCTGATAAACTCGTTGAACGAGGCACAAGCTGGAGATGTTGTACTTTTCCACGGGTGCTGTCACAACCCTACAGGAATAGATCCTACGCT<br>TGAACAATGGCAGACCTTAGCGCAATTGTCTGTTGAAAAAGGATGGTTACCGTTATTTGACTTTGCTTACCAGGGTTTTGCGCGCGGTCTGGAAGAGGATGCAGAGGGGCTGCG<br>GGCTTTTGCAGCTATGCATAAAGAACTTATAGTGGCCAGTAGCTACTCTAAAAATTTCGGGTTATACAACGAACGGGTCGGTGCGTGTACGTTGGTTGCAGCCGACTCTGAAACA<br>GTAGATCGTGCTTTCTCCCAAATGAAGGCAGCGATTAGAGCGAACTATTCTAATCCTCCCGCCCATGGCGCCTCGGTAGTAGCCACCATTCTTAGTAACGACGCCTTACGGGCCAT<br>ATGGGAACAAGAATTGACCGATATGCGCCAGCGGATTCAGAGAATGAGACAGCTGTTCGTTAACACGCTTCAAGAAAAGGGTGCCAACCGTGATTTCTCCTTTATTATCAAGCAG<br>AATGGCATGTTCTCATTTAGTGGGCTGACGAAAGAGCAAGTGCTGCGCCTGCGTGAGGAATTCGGTGTTTACGCAGTGGCTTCGGGACGTGTCAACGTGGCAGGAATGACACCG<br>GACAACATGGCCCCACTGTGTGAGGCAATTGTCGCCGTTCTGTAA |
| RBS2 | RBS4 | AAAGGAGAA |
| ipdC | Gene of interest 2 | ATGAGAACACCGTATTGCGTTGCAGATTACCTTTTAGATAGATTGACCGACTGTGGTGCAGATCACTTATTCGGGGTGCCTGGAGATTACAATTTACAGTTCTTAGACCATGTAAT<br>AGACTCCCCCGACATCTGTTGGGTCGGCTGTGCAAATGAATTGAACGCGAGCTACGCTGCAGACGGCTATGCGCGGTGTAAAGGGTTCGCCGCTCTTCTTACTACATTTGGCGTG<br>GGTGAACTTTCCGCAATGAATGGTATTGCGGGGTCTTATGCCGAGCACGTTCCAGTGCTGCATATAGTAGGCGCTCCGGGAACAGCGGCTCAACAGCGTGGGGAGTTACTTCAT<br>CATACCCTGGGCGATGGAGAATTTAGACACTTTTACCACATGAGCGAACCGATTACAGTTGCTCAAGCGGTTTTAACCGAGCAAAATGCCTGCTATGAAATTGATCGTGTACTTAC<br>AACCATGCTTCGTGAACGTAGACCTGGGTATTTGATGTTACCGGCCGATGTGGCGAAAAAGGCAGCTACTCCGCCTGTCAATGCCCTTACACACAAACAAGCGCATGCTGATTCG<br>GCGTGTCTTAAGGCTTTTCGCGACGCGGCTGAAAACAAATTAGCTATGTCTAAACGGACAGCGTTATTGGCCGACTTCTTAGTGTTGCGGCACGGCCTTAAACACGCATTACAGA<br>AGTGGGTAAAGGAAGTTCCTATGGCACACGCCACCATGTTGATGGGTAAAGGCATTTTTGATGAAAGACAGGCTGGGTTTTACGGTACTTACTCGGGTTCGGCCAGCACTGGTG<br>CAGTCAAAGAAGCCATAGAGGGGGCAGACACGGTCCTGTGCGTAGGTACCAGATTTACTGACACCCTGACTGCCGGCTTTACTCACCAATTAACACCTGCACAGACTATTGAGGT<br>CCAGCCGCACGCTGCCAGAGTGGGAGACGTTTGGTTCACTGGAATACCTATGAACCAGGCTATTGAGACACTGGTGGAACTTTGTAAGCAACATGTTCATGCAGGTCTTATGTCA<br>TCCTCGTCGGGGGCAATCCCCTTTCCCCAACCAGACGGTAGCCTTACCCAAGAGAACTTCTGGAGAACCCTGCAAACCTTCATACGCCCTGGCGATATCATCCTTGCGGACCAGG<br>GAACGTCAGCCTTCGGGGCTATAGACCTGCGGCTGCCTGCGGACGTTAATTTCATAGTACAGCCCCTTTGGGGATCAATTGGCTATACATTAGCTGCCGCGTTTGGGGCCCAAAC<br>AGCCTGTCCAAACCGGCGTGTTATTGTCCTGACAGGCGACGGGGCTGCTCAACTGACAATCCAGGAGCTTGGAAGCATGCTTAGAGACAAGCAACACCCGATCATCCTTGTGTTA<br>AACAACGAGGGCTACACCGTAGAACGGGCTATCCACGGCGCTGAGCAGCGCTACAACGATATCGCTTTATGGAATTGGACCCACATTCCGCAAGCCCTTTCACTGGACCCGCAAT<br>CGGAATGCTGGCGTGTCTCAGAAGCCGAACAATTGGCCGACGTACTGGAGAAGGTTGCCCATCACGAGCGTTTGAGTTTAATAGAAGTTATGCTGCCGAAGGCGGATATACCAC<br>CCTTGTTGGGAGCGCTGACGAAAGCGTTGGAGGCCTGCAATAACGCGTGA |
| RBS3 | RBS3 | ACAGGAGGG |
| iad1 | Gene of interest 3 | ATGCCTACACTGAACTTGGATTTGCCGAACGGCATAAAGAGTACGATCCAGGCAGACCTTTTTATAAATAATAAGTTTGTCCCCGCGTTGGATGGGAAGACGTTTGCAACGATTA<br>ACCCTAGTACCGGCAAGGAGATAGGCCAAGTCGCGGAGGCCTCGGCGAAAGACGTTGACTTAGCGGTGAAGGCCGCCCGTGAAGCTTTTGAGACTACTTGGGGCGAAAATACG<br>CCGGGAGACGCGCGGGGACGCTTGTTGATTAAATTAGCTGAATTGGTTGAGGCAAATATAGACGAATTAGCCGCAATCGAATCGCTGGATAACGGAAAAGCATTCTCCATTGCT<br>AAGTCCTTTGACGTGGCAGCCGTGGCCGCAAACTTAAGATATTATGGCGGTTGGGCAGATAAGAATCATGGCAAGGTGATGGAAGTTGACACGAAAAGATTGAATTATACTCGC<br>CACGAACCGATAGGTGTTTGTGGGCAAATCATTCCGTGGAACTTCCCACTGCTTATGTTCGCTTGGAAATTGGGCCCAGCCTTAGCCACGGGTAATACTATAGTTTTGAAAACGGC<br>AGAACAGACGCCCTTATCAGCAATCAAAATGTGTGAGCTTATAGTAGAAGCAGGTTTTCCTCCTGGGGTAGTGAACGTCATATCTGGCTTTGGACCTGTGGCGGGCGCGGCAATT<br>AGTCAGCATATGGACATCGATAAAATTGCCTTCACAGGTTCCACCTTAGTCGGTCGTAACATTATGAAAGCGGCAGCTAGCACGAATCTGAAAAAAGTGACCCTTGAGTTGGGTG<br>GGAAAAGCCCTAATATAATCTTCAAAGATGCAGACTTGGATCAAGCCGTTAGATGGAGTGCATTTGGCATTATGTTTAACCACGGGCAGTGTTGTTGTGCCGGTTCCCGCGTCTAC<br>GTCGAAGAAAGTATTTACGATGCCTTTATGGAGAAGATGACTGCCCATTGCAAGGCTTTACAGGTAGGAGATCCTTTCTCGGCCAATACGTTCCAGGGCCCTCAGGTGTCGCAGT<br>TACAGTACGACCGGATTATGGAATATATAGAATCTGGCAAGAAAGATGCCAACCTTGCATTAGGTGGGGTCAGAAAAGGGAACGAGGGCTATTTTATTGAACCAACTATCTTTAC<br>CGATGTTCCGCATGATGCCAAAATCGCAAAGGAAGAGATCTTCGGACCGGTTGTCGTTGTATCAAAATTTAAGGACGAAAAAGATTTGATTAGAATAGCTAACGATAGCATTTAC<br>GGATTAGCTGCGGCTGTGTTTAGTCGGGATATCTCACGCGCTATTGAGACGGCACATAAGCTTAAGGCCGGTACTGTATGGGTTAACTGTTATAACCAACTTATACCCCAAGTCCC<br>ATTCGGTGGCTACAAGGCCAGTGGGATTGGACGCGAGCTGGGTGAATATGCTCTGTCGAACTACACGAATATCAAGGCTGTACATGTAAACTTAAGTCAGCCTGCCCCAATTTAA |
| Terminator | Terminator | AAAGGGGCTACCGGCGAACCAGCAGCCCCTTTATAAAGGCGCTTCAGTAGTCAGACCAGCATCAGTCCTGAAAAGGCGGGCCTGCGCCCGCCTCCAGG |
| KanR | Kanamycin selection marker | ATGATTGAACAGGATGGCCTGCATGCGGGTAGCCCGGCAGCGTGGGTGGAACGTCTGTTTGGCTATGATTGGGCGCAGCAGACCATTGGCTGCTCTGATGCGGCGGTGTTTCGT<br>CTGAGCGCGCAGGGTCGTCCGGTGCTGTTTGTGAAAACCGATCTGAGCGGTGCGCTGAACGAGCTGCAGGATGAAGCGGCGCGTCTGAGCTGGCTGGCCACCACCGGTGTTCC<br>GTGTGCGGCGGTGCTGGATGTGGTGACCGAAGCGGGCCGTGATTGGCTGCTGCTGGGCGAAGTGCCGGGTCAGGATCTGCTGTCTAGCCATCTGGCGCCGGCAGAAAAAGTGA<br>GCATTATGGCGGATGCCATGCGTCGTCTGCATACCCTGGACCCGGCGACCTGTCCGTTTGATCATCAGGCGAAACATCGTATTGAACGTGCGCGTACCCGTATGGAAGCGGGCCT<br>GGTGGATCAGGATGATCTGGATGAAGAACATCAGGGCCTGGCACCGGCAGAGCTGTTTGCGCGTCTGAAAGCGAGCATGCCGGATGGCGAAGATCTGGTGGTGACCCATGGTG<br>ATGCGTGCCTGCCGAACATTATGGTGGAAAATGGCCGTTTTAGCGGCTTTATTGATTGCGGCCGTCTGGGCGTGGCGGATCGTTATCAGGATATTGCGCTGGCCACCCGTGATAT<br>TGCGGAAGAACTGGGCGGCGAATGGGCGGATCGTTTTCTGGTGCTGTATGGCATTGCGGCACCGGATAGCCAGCGTATTGCGTTTTATCGTCTGCTGGATGAATTTTTCTAA |
| Hok/Sok locus | Plasmid stability | ACAACATCAGCAAGGAGAAAGGGGCTACCGGCGAACCAGCAGCCCCTTTATAAAGGCGCTTCAGTAGTCAGACCAGCATCAGTCCTGAAAAGGCGGGCCTGCGCCCGCCTCCA<br>GGTTGCTACTTACCGGATTCGTAAGCCATGAAAGCCGCCACCTCCCTGTGTCCGTCTCTGTAACGAATCTCGCACAGCGATTTTCGTGTCAGATAAGTGAATATCAACAGTGTGAG<br>ACACACGATCAACACACACCAGACAAGGGAACTTCGTGGTAGTTTCATGGCCTTCTTCTCCTTGCGCAAAGCGCGGTAAGAGGCTATCCTGATGTGGACTAGACATAGGGATGCC<br>TCGTGGTGGTTAATGAAAATTAACTTACTACGGGGCTATCTTCTTTCTGCCACACAACACGGCAACAAACCACCTTCACGTCATGAGGCAGAAAGCCTCAAGCGCCGGGCACATC<br>ATAGCCCATATACCTGCACGCTGACCACACTCACTTTCCCTGAAAATAATCCGCTCATTCAGACCGTTCACGGGAAATCCGTGTGATTGTTGCCGCATCACGCTGCCTCCCGGAGTT<br>TGTT |

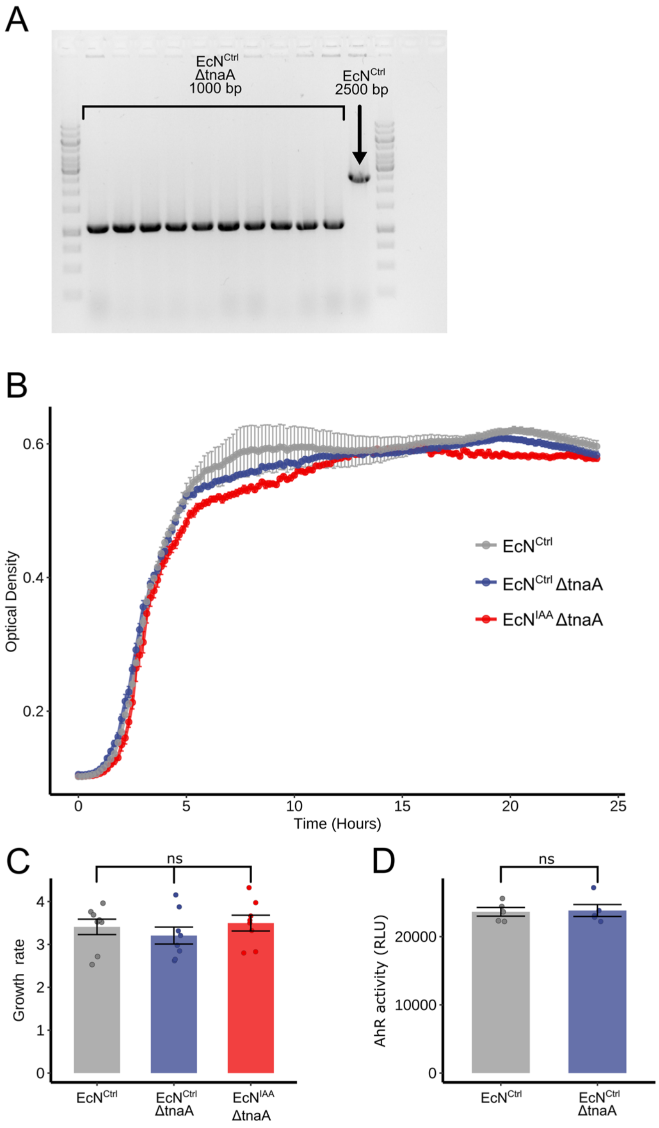


**Figure EV2. Confirmation of ΔtnaA knockout and functional characterization of *Escherichia coli* Nissle 1917 strains.**

(**A**) Agarose gel electrophoresis confirming the tnaA gene knockout in EcN. The wild-type control strain ($EcN^{Ctrl}$) shows a band of approximately 2500 bp, whereas the ΔtnaA knockout strain show a smaller band around 1000 bp, confirming successful deletion of the tnaA gene. (**B**) Growth curves of EcN strains over 24 h, measured by optical density at 600 nm ($n = 8$ biological replicates). Deletion of the tnaA gene does not significantly affect the growth rates of the strains. (**C**) Specific growth rate calculated from growth curves in (**B**). (**D**) AhR activity as relative luminescent units (RLU) derived from luciferase-expressing AhR reporter cells following stimulation with 10% supernatant of $EcN^{Ctrl}$ and $EcN^{Ctrl}$ ΔtnaA for 48 h ($n = 5$ biological replicates). Data are mean ± SEM. Statistical significance was determined with ANOVA and post hoc comparison analysis between groups using Tukey's honest significant difference test. Source data are available online for this figure.


© The Author(s)

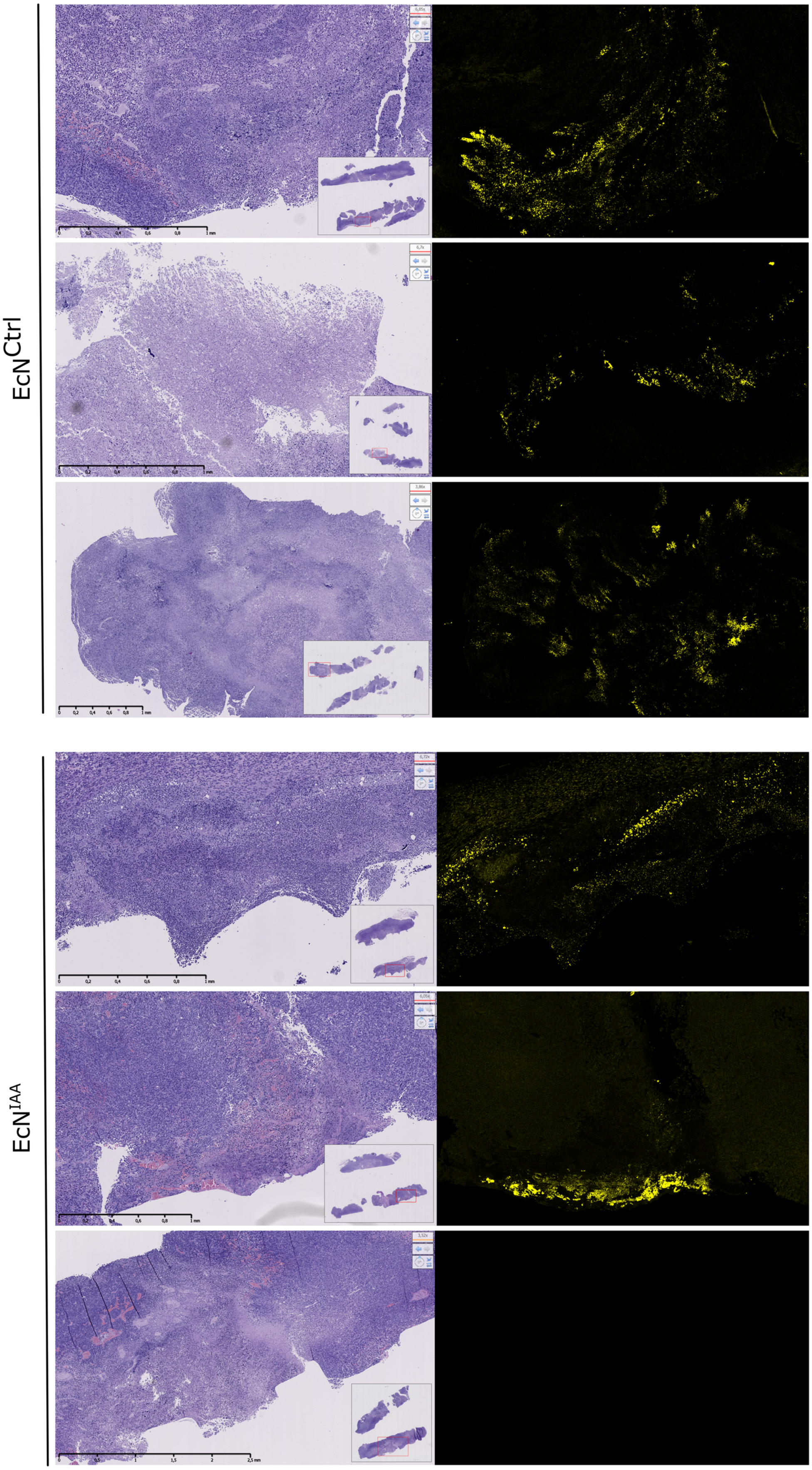
EcNCtrl
EcNIAA

**Figure EV3. Localization of EcN in necrotic tumor regions.**

Representative images of areas with necrosis and co-localized bacteria from animal study with CT26 tumors receiving an intratumoral injection of $EcN^{Ctrl}$ (top) and $EcN^{IAA}$ (bottom). Tumors of comparable size from $EcN^{IAA}$ ($n = 3$ biological replicates) and $EcN^{Ctrl}$ ($n = 3$ biological replicates) animals were analyzed in formalin-fixed paraffin-embedded sections using H&E staining (left column) and fluorescence in situ hybridization microscopy to detect bacteria on the adjacent slide (right column).

 © The Author(s)

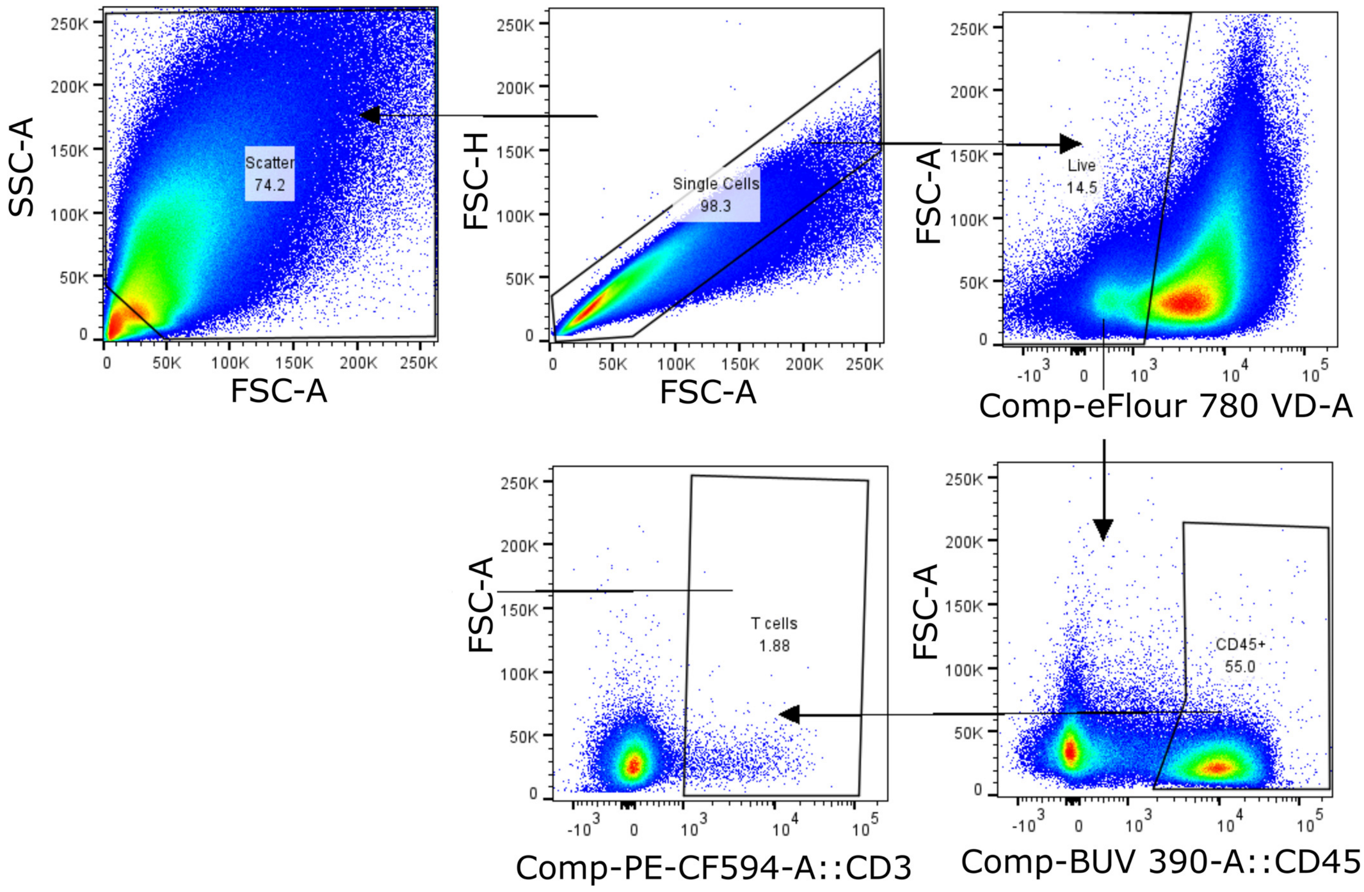


**Figure EV4. Gating strategy for T-cell panel related to Fig. 2g.**

Identification of viable T cells was defined as viability dye (VD), CD45 +, CD3 +. Fluorescence Minus One control was used to determine gates.
